# Case of Spontaneous Rupture of the Uterus in the Third or Fourth Month of Pregnancy

**Published:** 1846-09

**Authors:** 


ART. III.—*Case of spontaneous rupture of the Uterus in the third or fourth month of pregnancy. Congenital absence of muscular wall of Uterus on one side, of Fallopian tube and ovary, and of the os Tincæ. Communicated in a letter to Prof. Hamilton, by* ——— ———, M. D.*

———, Aug. 7, 1846.

DEAR SIR:

I have recently made an autopsy which I think of sufficient interest to report to you.

Miss. ——— aet. 17; was seized suddenly with pains resembling colic, accompanied with vomiting and faintness. A neighbor being called in prescribed as for colic, and the girl partially revived, but soon became faint again, and died.

*Autopsy, 24 hours after decease.*—Cavity of abdomen filled with coagulated blood, surrounding a fœtus of the third or fourth month. Uterus ruptured along its right side, from the fundus to near the neck. Left ovary and Fallopian tube entirely wanting. Right ovary and Fallopian tube were present, but the latter entered the uterus nigh to the neck, and the ovary was correspondingly lower than natural.

The uterus appeared to be developed to the size of the third or fourth month of gestation. The placenta was attached near the fundus upon the left side. On the side of the uterus where the rupture occurred, the walls of the organ were extremely thin. At the time of rupture there seemed to be nothing but the peritoneum, and in the immediate neighborhood the friability was such, that it was readily torn by the fingers with slight force. On the left side the walls were of the usual thickness, but did not present the common fibrous appearance. On examination of the neck no aperture could be found, nor was there any trace of an *os tincæ*. The neck resembled a tendon in appearance, but of less density. There was no communication whatever between the cavity of the uterus and the vagina.

I have retained the parts, and will embrace some opportunity to submit them to your inspection. The case is certainly one of interest as connected with the inquiry how the spermatic secretion

* For further account of this interesting case, see editorial department of the present number.—ED.

was transmitted so as to have effected fecundation. It would seem that it disproves incontestibly the necessity of the reception of this secretion into the uterine cavity, and from thence into the Fallopian tube; but the question remains, how *was* the necessary contact accomplished? I see no other method of explanation except by the old doctrine of absorption.

With much esteem, I remain yours,

Dr. F. H. Hamilton. ——— ———.

